# Metabolomic Profile and Antioxidant/Anti-Inflammatory Effects of Industrial Hemp Water Extract in Fibroblasts, Keratinocytes and Isolated Mouse Skin Specimens

**DOI:** 10.3390/antiox10010044

**Published:** 2021-01-01

**Authors:** Viviana di Giacomo, Lucia Recinella, Annalisa Chiavaroli, Giustino Orlando, Amelia Cataldi, Monica Rapino, Valentina Di Valerio, Matteo Politi, Marco Daniel Antolini, Alessandra Acquaviva, Francesco Bacchin, Massimo Di Mascio, Sheila Leone, Luigi Brunetti, Luigi Menghini, Simone Carradori, Gokhan Zengin, Gunes Ak, Claudio Ferrante

**Affiliations:** 1 Department of Pharmacy, Università Degli Studi “Gabriele d’Annunzio”, Via dei Vestini 31, 66100 Chieti, Italy; viviana.digiacomo@unich.it (V.d.G.); lucia.recinella@unich.it (L.R.); annalisa.chiavaroli@unich.it (A.C.); amelia.cataldi@unich.it (A.C.); matteo.politi@unich.it (M.P.); marcodaniel.antolini@studenti.unich.it (M.D.A.); alessandra.acquaviva@studenti.unich.it (A.A.); sheila.leone@unich.it (S.L.); luigi.brunetti@unich.it (L.B.); luigi.menghini@unich.it (L.M.); simone.carradori@unich.it (S.C.); claudio.ferrante@unich.it (C.F.); 2 Genetic Molecular Institute of CNR, Unit of Chieti, “G. d’ Annunzio” University, Via dei Vestini 31, 66100 Chieti, Italy; m.rapino@unich.it; 3 Department of Medicine and Ageing Sciences, “G. d’ Annunzio” University, Via dei Vestini 31, 66100 Chieti, Italy; valentina.divalerio@unich.it; 4 Veridia Italia Srl, Via Raiale 285, 65100 Pescara, Italy; f.bacchin@veridia.it (F.B.); massimo.dimascio96@gmail.com (M.D.M.); 5 Department of Biology, Science Faculty, Selcuk University, 42130 Konya, Turkey; akguneselcuk@gmail.com

**Keywords:** industrial hemp, keratinocytes, apoptosis, antioxidant/anti-inflammatory, bioinformatics, HPLC analysis, tyrosinase, indoleamine-2,3-dioxygenase, skin disorder(s), IL-6

## Abstract

Industrial hemp is a multiuse crop whose phytocomplex includes terpenophenolics and flavonoids. In the present study, the phenolic and terpenophenolic compounds were assayed in the water extract of the hemp variety Futura 75. Protective effects were also investigated in human fibroblast and keratinocytes and isolate mouse skin specimens, which were exposed to hydrogen peroxide and/or to the extract (1–500 µg/mL). The results of phytochemical analysis suggested the cannabidiol, cannabidiolic acid and rutin as the prominent phytocompounds. In the in vitro system represented by human keratinocytes and fibroblasts, the hemp extract was found to be able to protect cells from cytotoxicity and apoptosis induced by oxidative stress. Moreover, modulatory effects on IL-6, a key mediator in skin proliferation, were found. In isolated rat skin, the extract reduced hydrogen peroxide-induced l-dopa turnover, prostaglandin-E2 production and the ratio kynurenine/tryptpophan, thus corroborating anti-inflammatory/antioxidant effects. The in silico docking studies also highlighted the putative interactions between cannabidiol, cannabidiolic acid and rutin with tyrosinase and indoleamine-2,3-dioxygenase, involved in l-dopa turnover and tryptophan conversion in kynurenine, respectively. In conclusion, the present findings showed the efficacy of hemp water extract as a skin protective agent. This could be partly related to the extract content in cannabidiol, cannabidiolic acid and rutin.

## 1. Introduction

Skin, hair and eyes pigmentation are related to melanin synthesis which, in the skin, takes place in the epidermis [1]. From this tissue, the melanin is transferred via melanosomes to skin keratinocytes [2]. In physiological conditions, pigmentation plays a key role in skin protection, especially towards UV-induced injury [3]. However, the hyperpigmentation induced by excessive melanin production is involved in different skin disorders, including senile lentigo, acne, melasma and freckles [1]. Tyrosinase is a key enzyme involved in the melanogenesis process. In this pathway, tyrosinase converts tyrosine in l-dopa and the latter in dopaquinone. Therefore, the activity of tyrosinase can be evaluated as l-dopa turnover and/or dopaquinone production [4,5]. Tyrosinase can be regarded as the most prominent and successful target for melanogenesis inhibitors that directly inhibit the enzyme catalytic activity [6]. Consequently, enzyme inhibition could be crucial to counteract inflammatory-induced skin hyperpigmentation, especially during aging [7]. Natural compounds and herbal extracts have long been investigated for their antityrosinase activity [3]. Polar extracts displayed valuable capability in inhibiting directly the enzyme activity, displaying IC_50_ values in the micromolar range [5]. This effect is due, albeit partially, to the high content of phenolic compounds, often found in water and hydroalcoholic extracts from good yields. Furthermore, the use of water and hydroalcoholic solutions, especially those prepared via traditional methods including decoction and infusion, proved effective in conjugating pharmacological efficacy and safety. If the investigation of polar extracts is extended to plant materials traditionally considered as byproducts, innovative scenarios could be open for the improvement of the whole botanical chain [8,9]. Industrial hemp (*Cannabis sativa*) is a multiuse crop that has been widely cultivated throughout history as a valuable source of nutrients and fibers from stem, rather than for its content in ∆9-tetrahydrocannabinol (THC). Although fiber and seed fractions are hemp main products, there is a great deal of interest in extraction of hemp secondary metabolites, including terpenes, terpenophenolics and flavonoids which could be at the basis of health-promoting effects [10]. In this regard, hemp essential oil showed promising antimicrobial activities, whereas the whole plant decoction displayed pain reliever effects against migraine attack [11]. By contrast, the chemical composition and the biological activity from aqueous fraction obtained from industrial hemp female flowers have been so far poorly investigated. Considering that one of the main perspectives of industrial hemp chain production is the valorization of the use of the female inflorescence, to date, considered as waste material in the botanical chain of hemp fiber production, the aim of the present study was to investigate the skin protective effects induced by the water extract from the female inflorescences of the certified Futura 75 variety. The rationale of the investigation derives from the inhibitory effects induced by Futura 75 water extract on dermatophyte growth and tyrosinase activity [12]. The same extract was also effective in modulating peripheral tissue levels of serotonin (5-HT) and kynurenine metabolites, thus suggesting pro-homeostatic effects on tryptophan catabolism, whose increase has been related to skin inflammation [13]. In this context, a multidirectional investigation was conducted on fibroblasts, keratinocytes and isolated mouse skin specimens challenged with hydrogen peroxide, in order to trigger oxidative stress and to induce tyrosinase activity [14], and then exposed to different concentrations of Futura 75 water extracts. In the ex vivo biological model, the tyrosinase activity and the changes in prostaglandin E_2_ (PGE_2_) production were evaluated following extract treatment. Additionally, the kynurenine/tryptophan (KYN/TRP) ratio was measured as an index of indolamine-2,3-dioxygenae (IDO-1) activity, which was found to be increased in a preclinical model of skin inflammation [13,15]. In the in vitro models of human keratinocytes and primary fibroblasts, the cytotoxicity and potential protective effects of the water extracts were investigated. Cell proliferation, apoptosis occurrence and gene expression and release of interleukin-6 (IL-6) were evaluated. A phytochemical investigation was also conducted to determine the levels of cannabinoid phytocompounds in the water extract, whereas in silico docking experiments were carried out to explore the putative affinities of the prominent phytocompounds present in the extract towards the two enzymes tyrosinase and IDO-1.

## 2. Materials and Methods 

### 2.1. Hemp Sample, Reagents and Standard Solutions

The plant material consists in female inflorescences of Cannabis sativa L cultivar “Futura 75”, a certified non-THC producing cultivar approved for industrial production, cultivated in Abruzzo Region (Italy), under controlled conditions, avoiding chemical additives. Female inflorescences were manually harvested from plants at full blooming state and then immediately dried in a ventilated oven (40 °C). Once they reached the constant weight, samples were clumsily chopped and stored in airtight plastic bags, in a dark and dry place at room temperature (22–24°C), before performing phytochemical and biological assays. Plant identity was confirmed botanically and morphologically by the co-author Prof. Luigi Menghini. Samples were kindly supplied by Hemp Farm Italia (Tortoreto (TE), Italy) during the cultivation season 2018. The product is sold by the company itself as industrial hemp, and is, therefore, certified for THC content results <0.2% *w*/*w*, according to the European Regulation EC no. 1124/2008—12 November 2008. Terpenophenolic compound standards were purchased from Focus Analytics Srl (Arcore (MB), Italy) and Comedical Srl (Trento, Italy). Phenolic compound standards were purchased from Sigma-Aldrich (Milano, Italy).

### 2.2. Extract Preparation

A dried cultivar sample was weighed (0.2 g) using a Precisa XT220A balance (Micro Precision Calibration Inc., Grass valley, CA, USA) in 50 mL Falcon tubes and then immediately homogenized together with the extraction solvent using a T25 digital Ultra-Turrax tissue homogenizer (IKA, Staufen, Germany) for 30 s at 10,000 g. This treatment partially uniformed the grain size so that a better extraction could be performed. Subsequently, ultrasound-assisted extraction (UAE) of the homogenate was carried out. Distilled water was used as extraction solvent to simulate the possible home-made use (decoction, infusion) of hemp inflorescences. The sample tube with the mixture was placed in a Trans-sonic T460 ultrasonic bath (Elma, Singen, Germany). The operative conditions for the extraction were optimized through response surface methodology (RSM) [16]. A four factor Box-Behnken design was defined to investigate the effects of parameters such as time, temperature, power and solid/liquid ratio on water UAE of industrial hemp inflorescences. The effects of independent variables were evaluated as total phenol content (TPC), total flavonoid content (TFC) and total tannin content (TTC). The operative conditions such as extraction method (UAE) and solvent (water) were selected on the basis of a previous study [17]. The range applied for selecting independent variables are detailed in Table 1.

According to the experimental design, thirty-one extractions were defined for coding variables at three levels (arbitrary units: −1, 0, 1). In order to limit the time-consuming effect of this phase, a selection of the optimal design was performed applying the criterion of D-optimality and limiting the number of runs to the arbitrary value of 20, as detailed in Table 2. The surface analysis and analysis of variance (ANOVA) to define and optimize the Box–Behnken experimental conditions were conducted through Minitab 16 software. 

Details about the analysis of response surface design, effects of critical parameters on extraction performance and prediction of optimal conditions for extraction are reported in Supplementary Materials. The optimized extraction conditions were: 60 mg dry material in 1 mL water; UAE at 80 °C for 25 min.

### 2.3. Colorimetric Determination of Bioactive Components

The colorimetric measurement of total phenolic, flavonoid and tannin levels was conducted according to a recent study [18]. Standards, namely, gallic acid (GA) for phenolics, rutin (RU) for flavonoids and tannic acid for tannins, were used to explain the results. The antiperoxidase activity was measured by monitoring the formation of the highly fluorescent resorufin, produced by the interaction of Amplex Red® reagent (Thermo Fischer Scientific, Monza, Italy) with H_2_O_2_ in the presence of horseradish peroxidase (Sigma Aldrich), as described by Vargas and colleagues [19].

### 2.4. HPLC Analysis

The HPLC apparatus consisted of a two PU-2080 PLUS chromatographic pump, a DG-2080-54 line degasser, a mix-2080-32 mixer, UV, diode array (DAD) and detectors, a mass spectrometer (MS) detector (expression compact mass spectrometer (CMS), Advion, Ithaca, NY 14850, USA), an AS-2057 PLUS autosampler and a CO-2060 PLUS column thermostat (all from Jasco, Tokyo, Japan). Integration was performed by ChromNAV2 Chromatography software. Before injecting in the HPLC apparatus, hemp extract was centrifuged at 3500× *g* for 15 min, and supernatant diluted at 10 mg/mL.

### 2.5. HPLC-DAD-MS Determination of Phenolic Compounds

Futura 75 water extract was analyzed for phenol quantitative determination using a reversed-phase HPLC–DAD-MS in gradient elution mode, in agreement with literature data [20]. The separation was conducted within the 30 min of the chromatographic run, starting from the following separation conditions: 0.23% formic acid, 93% water, 7% methanol. The details about gradient are listed in Table 3. The separation was performed on Infinity lab Poroshell 120 reverse phase column (C18, 150 × 4.6 mm i.d., 2.7 µm) (Agilent Santa Clara, CA, USA). Column temperature was set at 30 °C.

Quantitative determination of phenolic compounds was performed via DAD detector. The extract was also qualitatively analyzed with an MS detector in negative ion mode (vanillic acid, ferulic acid, naringenin) and positive ion mode (rutin). MS signal identification was realized through comparison with standard solutions and MS spectra present in the MassBank Europe database (https://massbank.eu/MassBank/). The list of compounds analyzed the wavelengths and the *m/z* ratio for their determination are listed in Table 4. Quantification was done through 7-point calibration curves, with linearity coefficients (R^2^) > 0.999, in the concentration range 2–140 µg/mL. The limits of detection were lower than 1 µg/mL for all assayed analytes. The area under the curve from HPLC chromatograms was used to quantify the analyte concentrations in the extract.

### 2.6. HPLC-UV-MS Determination of Terpenophenolic Compounds

Futura 75 water extract was analyzed for terpenophenol quantitative determination using a reversed-phase HPLC–UV-MS in gradient elution mode. HPLC: the separation was conducted within 30 min, starting from the following conditions: 0.007% formic acid, 7% water, 93% acetonitrile. The details about gradient are listed in Table 5. The separation was performed on an Infinity lab Poroshell 120 reverse phase column (C18, 150 mm × 4.6 mm i.d., 2.7 µm) (Agilent Santa Clara, CA, USA). Column temperature was set at 30 °C.

The extract was qualitatively analyzed with MS detector in positive ion mode. MS signal identification was realized through comparison with standard solutions and MS spectra present in the MassBank Europe database (https://massbank.eu/MassBank/). Specifically, the mass to charge ratio (*m/z*), the wavelength and retention times considered for analyte identification are listed in Table 6.

Quantitative determination of phenolic compounds identified by MS analysis was performed via UV detector at 230 nm wavelength. Quantification was performed through 7-point calibration curves, with linearity coefficients (R^2^) > 0.99, in the concentration range 2–160 µg/mL. The area under the curve from HPLC chromatograms was used to quantify the terpenophenol concentration in the extract. The limits of detection were lower than 0.9 µg/mL for all assayed analytes. Regarding the terpenophenolic recovery, the plant material was subjected to 22 cycles of extraction at 80 °C for 25 min in order to extract all compounds, whose absence was confirmed by MS analyses. The obtained blank matrix was, therefore, added with standard solutions (6 µg/mL) of cannabinoids and subjected to a further extractive procedure. The obtained recoveries were in the range 85.61–94.51%.

### 2.7. Qualitative Analysis of Extract’s Cannabidiol Content Via Protonic Magnetic Resonance (^1^H-NMR) 

Protonic magnetic resonance (^1^H-NMR) analysis was conducted through a Varian 300 MHz spectrometer using standard proton pulse sequence (s2pul). Samples were prepared as follows: 10 mg/mL cannabidiol and 50 mg/mL extract were sonicated in CDCl_3_ for 30 min at room temperature. An amount of 600 µL of each sample was transferred into the NMR tube and analyzed with the following parameters: acquisition time: 1.706 s; width: 4803.1 Hz; number of scans: 128.

### 2.8. Cell Culture

Primary human gingival fibroblasts (HGFs) were obtained from fragments of healthy marginal gingival tissue taken from the retromolar area removed during surgical extraction of third molars in adult subjects following the regularization of the surgical flap before sutures. The tissue fragments were then processed as already reported [21]. The study was approved by the Ethics Committee of G. d’Annunzio University, Chieti (Italy) (prot. N° 1173 01.03.2016).

The human keratynocytes cell line HaCaT cells were purchased from American Type Culture Collection (ATCC, Manassas, VA, USA), and both cell types were maintained in a high glucose Dulbecco’s modified Eagle’s medium (DMEM) (EuroClone, Milan, Italy) supplemented with 10% fetal bovine serum (FBS) and 1% penicillin/streptomycin (both from Gibco, Life Technologies, Carlsbad, CA, USA) under humidified air composed of 5% CO_2_ at 37 °C.

### 2.9. MTT Assay

After reaching the exponential phase of growth, the cells were seeded into 96-well culture plates at a density of 8 × 10^3^ cells per well and let them to adhere for 24 h. Both HGFs and HaCaT cells were then washed and treated with 500 µM H_2_O_2_ for 4 h and different concentrations of water hemp extract (1–500 μg/mL) for 24, 48 and 72 h starting from the exposure to H_2_O_2_. Complete DMEM medium was used as a negative control.

At 24, 48 and 72 h, both HGFs and HaCat keratinocytes were labeled with MTT (3-(4,5-dimethylthiazol-2-yl)-2,5-diphenyltetrazolium bromide) solution (Sigma Aldrich, Milan, Italy) for 4 h at 37 °C, and the formazan product was solubilized with dimethyl sulfoxide (DMSO). The absorbance of the solutions was measured at 540 nm using a plate spectrophotometer (Multiskan GO, Thermo Fisher Scientific, Waltham, MA, USA).

### 2.10. Flow Cytometry Apoptosis Detection

A FITC Annexin-V apoptosis detection kit (BD Pharmingen, San Diego, CA, USA) was used to detect apoptosis, following the manufacturer’s instructions. Briefly, 10^5^ cells were gently re-suspended in 100 µL of binding buffer and incubated for 15 min at room temperature in the dark with 5 µL of Annexin-V-FITC and 5 µL of Propidium Iodide (PI). After the addition of 200 µL of binding buffer, samples were analyzed with a Cytoflex flow cytometer with the FL1 and FL3 detector in a log mode, using the Cytoexpert analysis software (both from Beckmann Coulter). For each sample, 10,000 events were collected.

### 2.11. IL-6 ELISA Assay

At each time point, the supernatants of HaCat cells were harvested, and the secretion of interleukin-6 (IL-6) in the culture media was evaluated by an ELISA kit (both from Enzo Life Sciences, Farmingdale, NY, USA), according to the manufacturer’s instructions. The optical density values were obtained by measuring the absorbance at 450 using a Multiscan GO microplate spectrophotometer (Thermo Fisher Scientific).

### 2.12. RNA Extraction, Reverse Transcription and Real-Time Reverse Transcription Polymerase Chain Reaction (Real-Time RT PCR)

Total RNA was extracted from the HaCaT cells using TRI Reagent (Sigma–Aldrich, Milano, Italy), according to the manufacturer’s protocol. Contaminating DNA was removed using 2 units of RNase-free DNase 1 (DNA-free kit, Ambion, Austin, TX, USA). The RNA concentration was quantified at 260 nm by spectrophotometer reading (BioPhotometer, Eppendorf, Hamburg, Germany), and its purity was assessed by the ratio at 260 and 280 nm readings. The quality of the extracted RNA samples was also determined by electrophoresis through agarose gels and staining with ethidium bromide, under UV light. One microgram of total RNA extracted from each sample in a 20 µL reaction volume was reverse transcribed using High Capacity cDNA Reverse Transcription Kit (Thermo Fisher Scientific, Waltham, MA, USA). Reactions were incubated in a 2720 Thermal Cycler (Thermo Fisher Scientific, Waltham, MA, USA) initially at 25 °C for 10 min, then at 37 °C for 120 min and finally at 85 °C for 5 s. Gene expression of IL-6 was determined by quantitative real-time PCR using TaqMan probe-based chemistry, as previously described [17]. PCR primers and TaqMan probes, including β-actin used as the housekeeping gene, were purchased from Thermo Fisher Scientific (Assays-on-Demand Gene Expression Products, Hs00174131_m1 for IL-6 gene, Hs99999903_m1 for β-actin gene). The real-time PCR was carried out in triplicate for each cDNA sample in relation to each of the investigated genes. Data were elaborated with the Sequence Detection System (SDS) software version 2.3 (Thermo Fisher Scientific, Waltham, MA, USA). Gene expression was relatively quantified by the comparative 2^−∆∆Ct^ method [22].

### 2.13. Ex Vivo Model of Hydrogen Peroxide-Induced Toxicity in Isolated Mouse Skin Tissue

Thirty-two male adult C57BL6 mice (20–25 g) were sacrificed by CO_2_ inhalation (100% CO_2_ at a flow rate of 20% of the chamber volume per min), and skin specimens were immediately collected from the back of the animals and maintained in a humidified incubator with 5% CO_2_ at 37 °C for 4 h, in DMEM buffer with the addition of hydrogen peroxide (1 mM). The experimental procedure was approved by the Italian Ministry of Health (Authorization Number: F4738.N.5QP) During the incubation period, skin specimens were stimulated with different concentrations of water hemp extract (1–500 μg/mL). Tissue supernatants were collected, and the PGE_2_ level (ng/mg wet tissue) was measured by radioimmunoassay (RIA), as previously reported [23]. L-dopa levels were quantified in the supernatants through high performance liquid chromatography (HPLC) coupled to electrochemical detection. The detailed protocol related to l-dopa identification and quantification is included in a previous article of ours [24], whereas the quantitative determination of tryptophan (TRP) and kynurenine (KYN) in the tissue medium was carried out on a reversed phase HPLC-fluorimeter in agreement with the method employed by Pocivavsek and colleagues [25]. In details, KYN and TRP analyses were performed by using a liquid chromatograph (MOD. 1525, Waters Corporation, Milford MA, USA) equipped with a fluorimetric detector (MOD. 2475, Waters Corporation), a C18 reversed-phase column (Kinetex^®^, 2.6 µm, 4.6 × 100 mm, Phenomenex, Torrance, CA, USA) and an on-line degasser (Biotech 4-CH degasi compact, LabService, Anzola Emilia, Italy). The separation was conducted in isocratic conditions, and the mobile phase consisted of 250 mM zinc acetate, 50 mM sodium acetate and 3% acetonitrile (pH adjusted to 6.2 with glacial acetic acid), using a flow rate of 1.0 mL/min. In the eluate, TRP and KYN were identified and measured fluorimetrically (KYN: excitation: 365 nm; emission: 480 nm; TRP: excitation: 285 nm; emission: 365 nm).

### 2.14. Bioinformatics

Docking calculations were conducted through the Autodock Vina of PyRx 0.8 software, as recently described [26]. Crystal structures of target proteins were derived from the Protein Data Bank (PDB) with PDB IDs as follows: 6KOF [indoleamine 2,3-dioxygenase (IDO-1)]; 5I3B [Tyrosinase]. A discovery studio 2020 visualizer was employed to investigate the protein–ligand non-bonding interactions.

### 2.15. Statistical Analysis

The experimental data related to in vitro and ex vivo studies were analyzed through the analysis of variance (ANOVA) followed by a Newman–Keuls post hoc test. The GraphPad Prism software was employed for the statistical analysis. *p* < 0.05 was considered statistically significant. The number of animals to be employed in the study was calculated using G*Power software (v3.1.9.4, University of Kiel, Kiel, Germany). The values of the study potency (1-β) and the significance level (α) were 0.8 and 0.05, respectively.

## 3. Results and Discussion

In the present study, the water extract from Futura 75 was assayed for the content of different classes of secondary metabolites, including phenols and terpenophenols. The formers were analyzed through colorimetric assays yielding total phenols, flavonoids, tannins and antiperoxidase activity (Table 7) that support the evaluation of antioxidant and anti-inflammatory effects in biological models.

**Figure 1:**
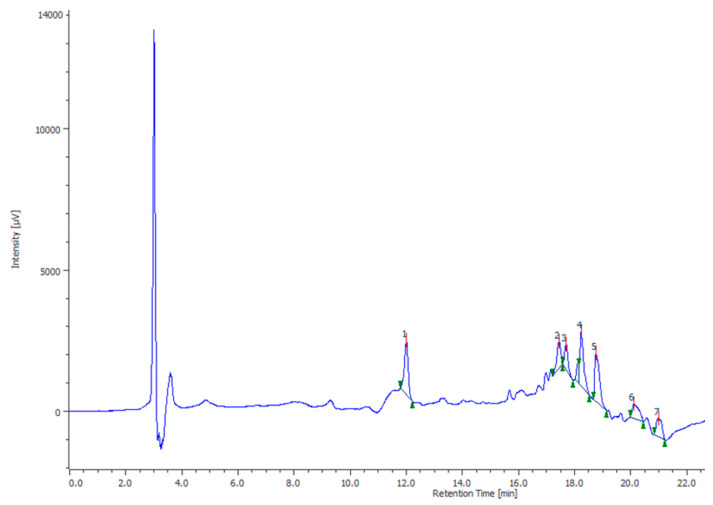
HPLC-DAD analysis of phenolics present in the water extract of Futura 75 hemp variety. Specifically, vanillic acid (2), ferulic acid (4), rutin (5) and naringenin (6) were identified through comparison with standard solutions.

**Figure 2:**
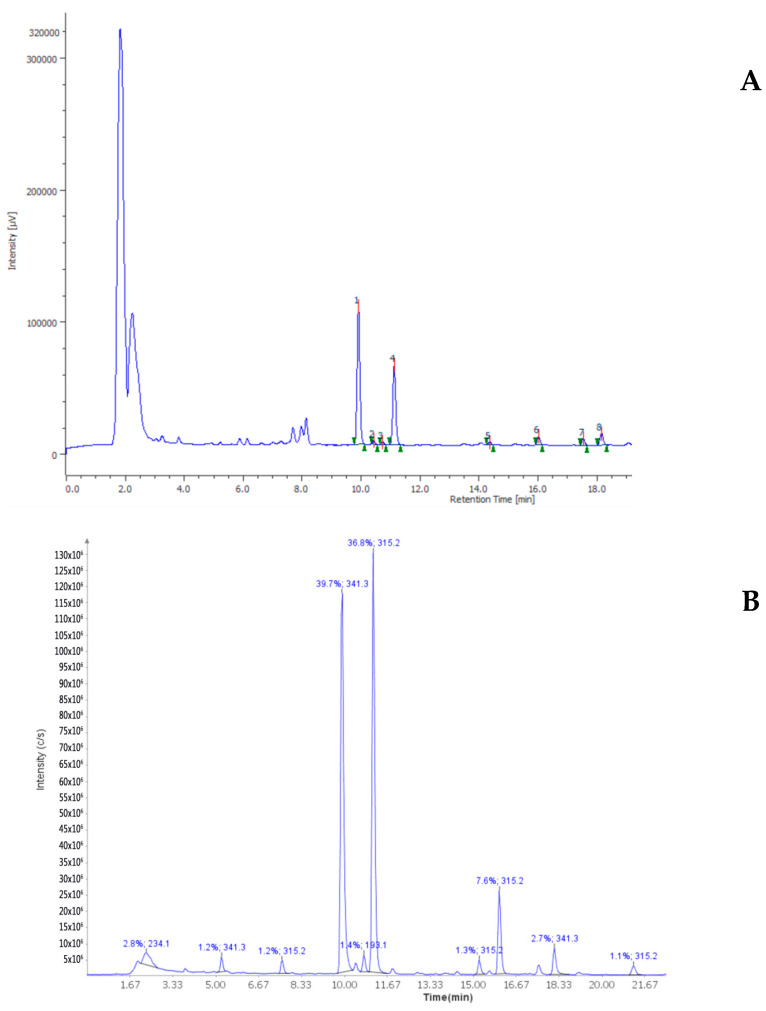
Chromatographic analysis of Futura 75 terpenophenols, in the water extract. (**A**) HPLC-UV chromatogram; (**B**) HPLC-MS chromatogram. In both subfigures cannabidiolic acid (1) and cannabidiol (4) areas are prominent compared to the other identified compounds.

**Figure 3:**
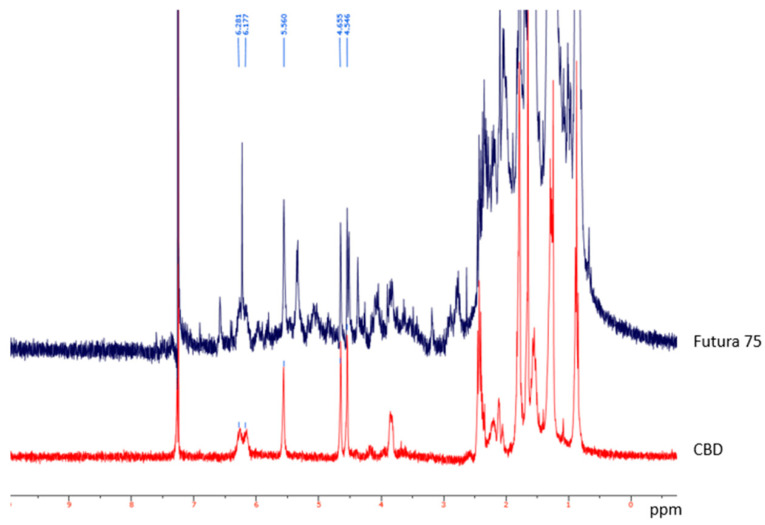
^1^H-NMR spectra of industrial hemp extract (**blue**) and cannabidiol standard solution (**red**). The cannabidiol protons signals are visible at 4.546, 4.655, 6.177 and 6.281 ppm and is detected in both cannabidiol standard and extract.

The HPLC-DAD also confirmed the presence of rutin, naringenin, ferulic acid and vanillic acid levels (Figure 1) that were consistent with the qualitative fingerprint analysis conducted on similar extracts prepared from Futura 75 plants harvested in 2017 [8,12]. Additionally, the levels of terpenophenols were determined through HPLC-UV-MS and ^1^H-NMR techniques. Specifically, the phytochemical analysis showed that hemp extract displayed a prominent quantity of cannabidiol (CBD) and its acid form (CBDA), compared to the other identified cannabinoids, including cannabigerol, cannabinol, cannabigerolic acid, THC and THCA (Figure 2). These results are also consistent with a recent paper by Nuapia and colleagues [27] showing a low ratio THCtotal/CBDtotal (0.18) in the water extract of industrial hemp seeds. The presence of CBD was also confirmed by ^1^H-NMR analysis. In this context, the water hemp spectrum was compared to CBD standard solution spectrum, giving, in particular, specific chemical shift signals at 4.546, 4.655 and 6.177 ppm, in CDCL_3_ (Figure 3), that are consistent with literature [28]. The phytochemical composition of the extract also agrees with antioxidant, anti-inflammatory and antimicrobial activities [8]. Additionally, the antimycotic effects against different dermatophytes species and the intrinsic antityrosinase effect [12] suggest potential applications against skin disorders, including hyperpigmentation [29,30].

The in vitro model for assessing the cytoprotective, antioxidant anti-inflammatory effects of the hemp water extract in skin consists in the human keratinocyte cell line HaCat and in primary human gingival fibroblast (HGFs). The epidermis is mainly constituted of keratinocytes, which are involved in the response of the skin to a variety of stimuli. In addition, fibroblasts strictly interact with keratinocytes and are involved in wound healing [31].

**Figure 4:**
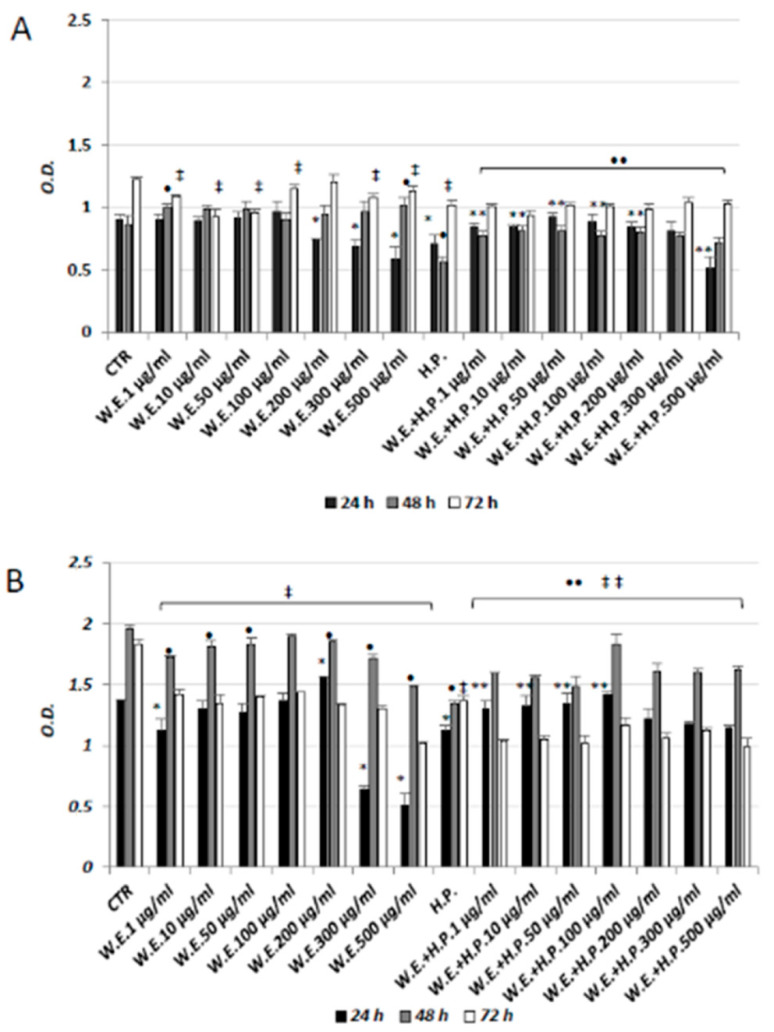
MTT assay in HGFs (**A**) and HaCat cells (**B**) in presence of different concentrations of hemp water extract (W.E.) and/or H_2_O_2_ (H.P.). n = 3. * *p* < 0.05 vs. Ctr 24 h; • *p* < 0.05 vs. Ctr 48 h; ‡ *p* < 0.05 vs. Ctr 72 h; ** *p* < 0.05 vs. H.P. 24 h; •• *p* < 0.05 vs. H.P. 48 h; ‡‡ *p* < 0.05 vs. H.P. 72 h.

Therefore, in the present study, different concentrations of Futura 75 water extract (ranging from 1 to 500 µg/mL) were administered to both cell types, HaCat and HGFs, representing the epithelial and connective components of the human skin, respectively. First, cell viability and proliferation were evaluated by MTT assay at three experimental points. The administration of the extract to HGFs (Figure 4A) exerted no remarkable effects, other than a decrease in cell proliferation especially for the concentrations higher than 100 µg/mL. On the other hand, when the cells are challenged with the hydrogen peroxide, their viability appears decreased, whereas the treatment with Futura 75 extract is able to increase cell metabolism both at 24 and 48 h at almost all the concentrations. As for HaCat cells, their viability seems affected by the exposure to the water extract when exposed to high concentrations (300 and 500 µg/mL) and longer experimental times (72 h) (Figure 4B). These findings are in line with recent literature in which the same cell line was treated with essential oil from female inflorescence [32]. Again, when cell proliferation is affected by H_2_O_2_ administration, the exposure to hemp extract at concentration up to 100 µg/mL exerted a protective effect both at 24 and 48 h.

Hemp seed hexane extract was recently found to be effective in modulating inflammation markers in both keratinocytes and fibroblasts in a model of a widely spread skin disorder, acne [33]. The phytochemical analysis suggests that cannabidiol and its acidic form are among the prominent phytocompounds in the extract from the industrial hemp variety used for this study. Although no effects of cannabidiolic acid were already reported in skin and skin cells, cannabidiol protective effects in dermatology are already investigated [34]. In particular cannabidiol was found to be able to activate antioxidant pathways both in fibroblasts and keratinocytes exposed to UV radiation and hydrogen peroxide [35,36] in accordance with the results from the MTT assay.

**Figure 5:**
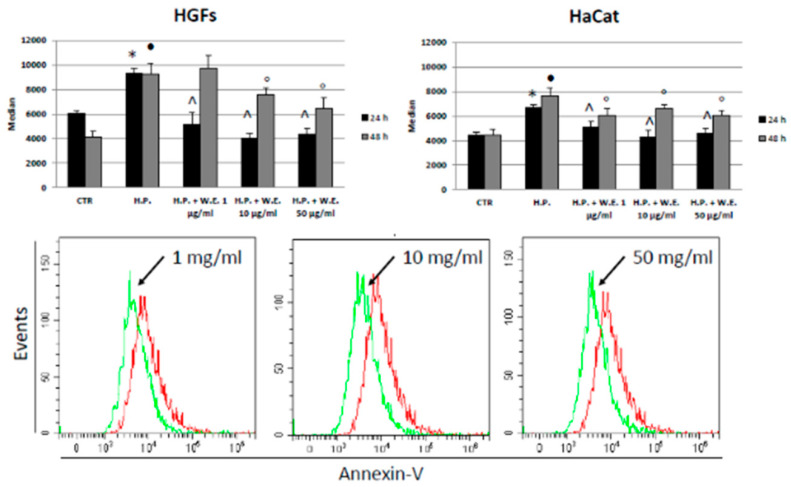
Apoptosis assay, expressed as the median of Annexin-V FITC peaks in HGFs (upper left) and HaCat cells (upper right) in presence of different concentrations of hemp water extract (W.E.) and/or H_2_O_2_ (H.P.) at 24 and 48 h. Representative histograms of HaCat cells at 24 h (below). Red line: H.P. treated cells. n = 3. * *p* < 0.05 vs. Ctr 24 h. • *p* < 0.05 vs. Ctr 48 h; ^ *p* < 0.05 vs. H.P. ° *p* < 0.05 vs. H.P. 48 h.

Basing on the previous results, a more focused analysis was performed on the lowest concentrations, namely, 1, 10 and 50 µg/mL, and earlier experimental times, i.e., 24 and 48 h, in order to better understand the mechanisms regulating the cellular response to the Futura 75 extract. Oxidative stress is known to trigger apoptosis, so the occurrence of programmed cell death was investigated in our experimental system (Figure 5). The exposure to hydrogen peroxide increases the apoptosis occurrence in both cell types and both experimental points (24 and 48 h). However, the water extract was able to decrease the number of apoptotic cells almost in all the conditions, even though no dose-related effect was registered. As for cannabidiol antiapoptotic effects in skin, there is only one study demonstrating the protective modulatory effect of CBD in anti- and pro-apoptotic factors in nude rats [37]. However, as the hemp extract rich in many components, not all its beneficial effects need to be addressed to CBD, as demonstrated by Sangiovanni and collaborators [38]. Rutin is the prominent flavonoid present in Futura 75 extract and, indeed, it was found to be already active in counteracting apoptosis either in keratinocytes or in fibroblasts [39].

**Figure 6:**
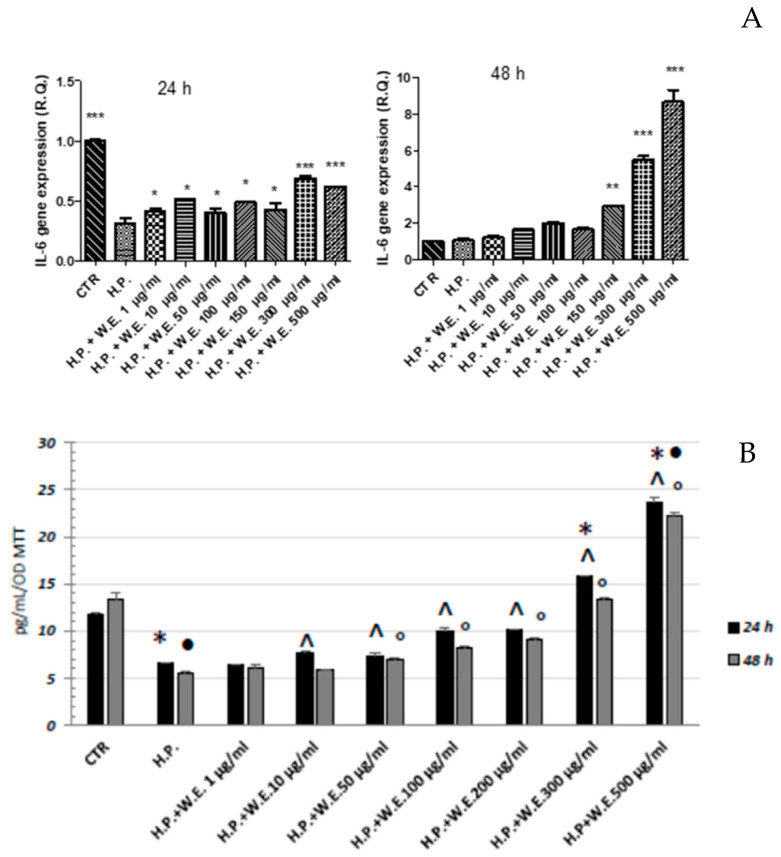
Modulatory effects induced by Futura 75 water extract on hydrogen peroxide (H.P.) 300 µM gene expression (**A**) and release (**B**) of interleukin (IL)-6, in human keratinocytes. n = 3. ANOVA, *p* < 0.0001; *** *p* < 0.001, ** *p* < 0.01, * *p* < 0.05 vs. respective H.P. group B * *p* < 0.05 vs. Ctr 24 h; • *p* < 0.05 vs. Ctr 48 h; ^ *p* < 0.05 vs. H.P. 24 h; ° *p* < 0.05 vs. H.P. 48 h.

Keratinocytes are involved in the initiation and perpetuation of skin inflammatory response releasing several proinflammatory mediators, including interleukin-6, under inflammation stimuli [40]. In the attempt to better investigate the occurrence of inflammation in the epithelial component of the skin, the gene expression and release of the interleukin-6 were then evaluated in the keratinocyte cell line (Figure 6). This cytokine was chosen because it is a key modulator of the inflammatory and reparative process in skin: it is involved in the differentiation, activation, and proliferation of leukocytes, endothelial cells, keratinocytes and fibroblasts. In addition to a pro-inflammatory function, IL-6 plays a role also in angiogenesis, granulation tissue stimulation and mitogenesis [41]. The IL-6 production was decreased after the administration of H_2_O_2_, in accordance with the reduced gene expression. The exposure to different concentrations of hemp water extract for 24 h seems to slightly increase the IL-6 gene expression (Figure 6A) and release (Figure 6B) up to the concentration of 200 µg/mL. The highest concentrations, on the other hand, induced a release of IL-6 at levels higher than the control sample. After 48 h of treatment, the high IL-6 production of the cells exposed to the highest concentration of Futura 75 is mirrored by the gene expression which was increased with respect to the control sample. Summarizing, the exposure to H_2_O_2_ disturbed keratinocyte proliferation and IL-6 gene expression and production. The administration of Futura 75 extract proved to be able to restore, albeit partially, the levels of this key cytokine in skin physiology [42].

**Figure 7:**
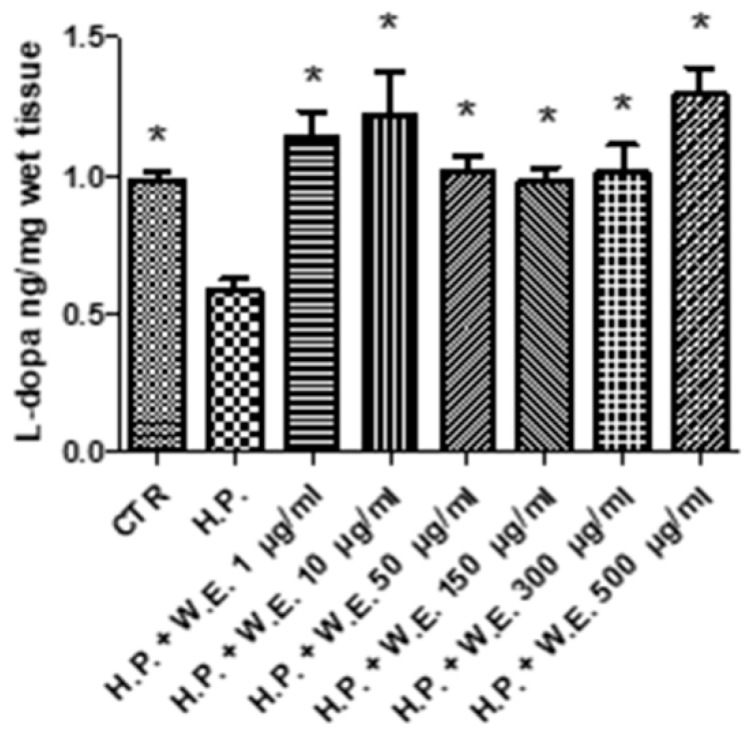
Inhibitory effect induced by Futura 75 water extract (W.E.) on L-dopa turnover increased by hydrogen peroxide (H.P.) 1 mM in isolated mouse skin specimens. ANOVA, *p* < 0.01; * *p* < 0.05 vs. H.P.

**Figure 8:**
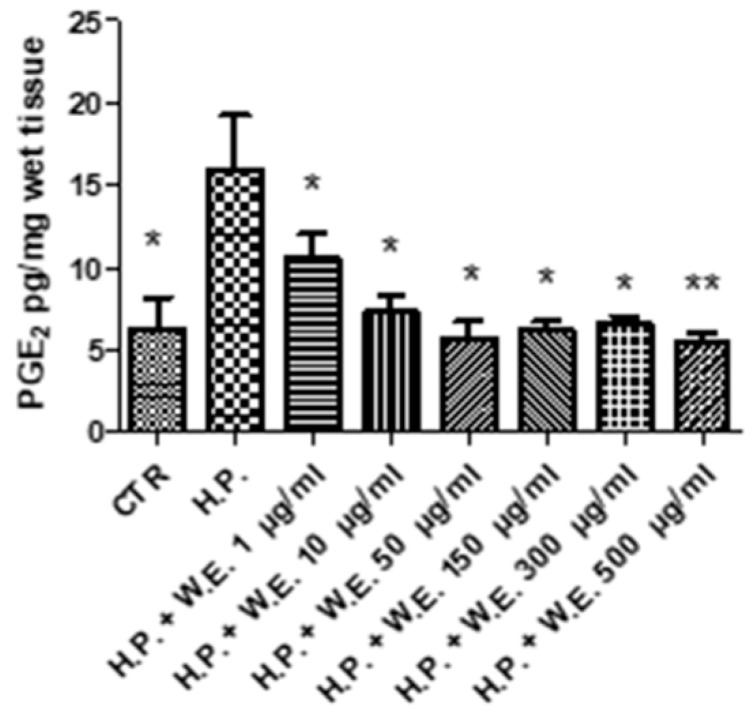
Inhibitory effect induced by Futura 75 water extract (W.E.) on prostaglandin (PG)E_2_ production increased by hydrogen peroxide (H.P.) 1 mM, in isolated mouse skin specimens. ANOVA, *p* < 0.01; ** *p* < 0.01, * *p* < 0.05 vs. H.P.

The water extract of Futura 75 was tested in isolated skin tissues challenged with hydrogen peroxide (1 mM), an ex vivo experimental model able to induce oxidative stress burden and tyrosinase activity [5]. Specifically, the extract 1–500 µg/mL was able to blunt the hydrogen peroxide-induced l-dopa turnover (Figure 7) that is closely related to the upregulation of tyrosinase activity [4,5]. The reduction in l-dopa turnover induced by Futura 75 extract is consistent with our recent finding of reduced intrinsic antityrosinase activity by water hemp extract l [12]. Since tyrosinase is a crucial enzyme involved in the synthesis of melanin through melanogenesis, the inhibition of its catalytic activity can be regarded as the most prominent and successful target for melanogenesis inhibition [6]. Additionally, the Futura 75 extract was able to reduce the PGE_2_ production (Figure 8), although at the highest concentrations, thus confirming the anti-inflammatory effects also observed in keratinocytes. On one side, this could depend on selective inhibition of cyclo-oxygenase-2 induced by the phenolic and terpenophenolic compounds, identified in the extract [43,44]. However, we cannot exclude that the reduction in PGE_2_ could be related, albeit partially, to the total phenol content and the related scavenging/reducing activity [45].

**Figure 9:**
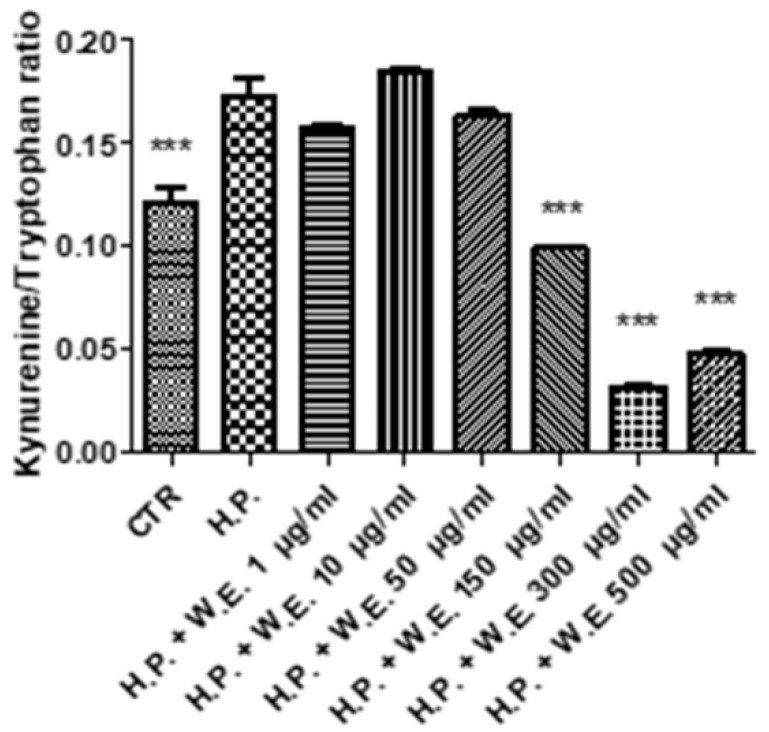
Inhibitory effect induced by Futura 75 water extract (W.E.) on kynurenine/tryptophan ratio increased by hydrogen peroxide (H.P.) 1 mM in isolated mouse skin specimens. ANOVA, *p* < 0.0001; *** *p* < 0.001 vs. H.P.

The Futura 75 extract was also capable of contrasting the hydrogen peroxide-induced changes in the KYN/TRP ratio (Figure 9). The latter has been considered as a valuable index for measuring the activity of IDO-1 that is recognized as the rate limiting enzyme in the degradative tryptophan pathway [46]. Recent studies showed the involvement of this enzyme in skin inflammation [13,15], thus suggesting the IDO-1 inhibition as an innovative pharmacological target in skin disorders. Plant secondary metabolites, including phenols and terpenophenols, have been reported to exert antioxidant and enzyme inhibitory effects [47,48]. High correlation also exists between radical scavenging and enzyme inhibition [49]. However, docking studies suggest selective inhibitory effects induced by plant secondary metabolites on both tyrosinase [50] and IDO-1 [51].

**Figure 10:**
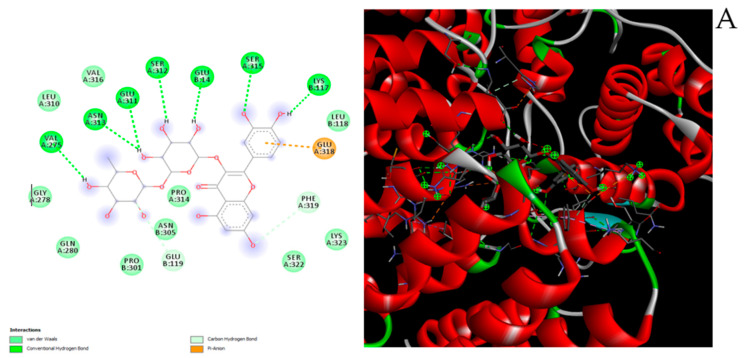

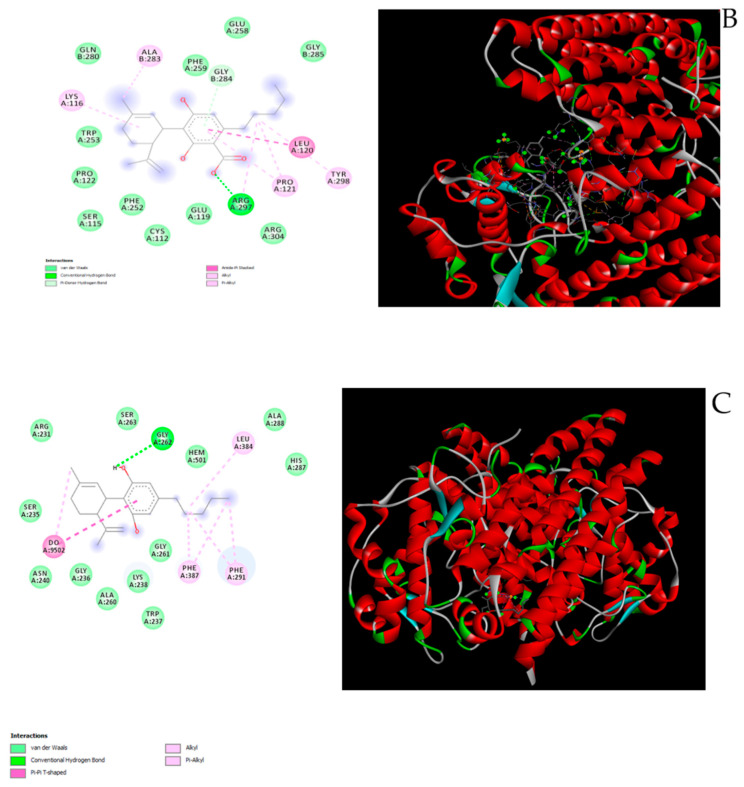
(**A**) Putative interactions between rutin and indoleamine-2,3-dioxygenase (IDO-1; PDB: 6KOF). Free energy of binding (ΔG) and affinity (Ki) are −9.2 kcal/mol and 0.2 µM, respectively; (**B**) putative interactions between cannabidiolic acid and indoleamine-2,3-dioxygenase (IDO-1; PDB: 6KOF). Free energy of binding (ΔG) and affinity (Ki) are −7.6 kcal/mol and 2.7 µM, respectively. (**C**) Putative interactions between cannabidiol and indoleamine-2,3-dioxygenase (IDO-1; PDB: 6KOF). Free energy of binding (ΔG) and affinity (Ki) are −7.4 kcal/mol and 3.8 µM, respectively.

Considering the results of phytochemical analysis, suggesting cannabidiol, cannabidiolic acid and rutin as the prominent phytocompounds in the extract, docking runs were performed to predict their putative affinities towards the two enzymes tyrosinase and IDO-1. Regarding the latter, all tested compounds displayed an affinity in the submicromolar/micromolar range (Figure 10A–C), thus suggesting that the observed reduction in KYN/TRP ratio following extract treatment could be related to specific interactions between the aforementioned hemp phytocompounds and the enzyme.

**Figure 11:**
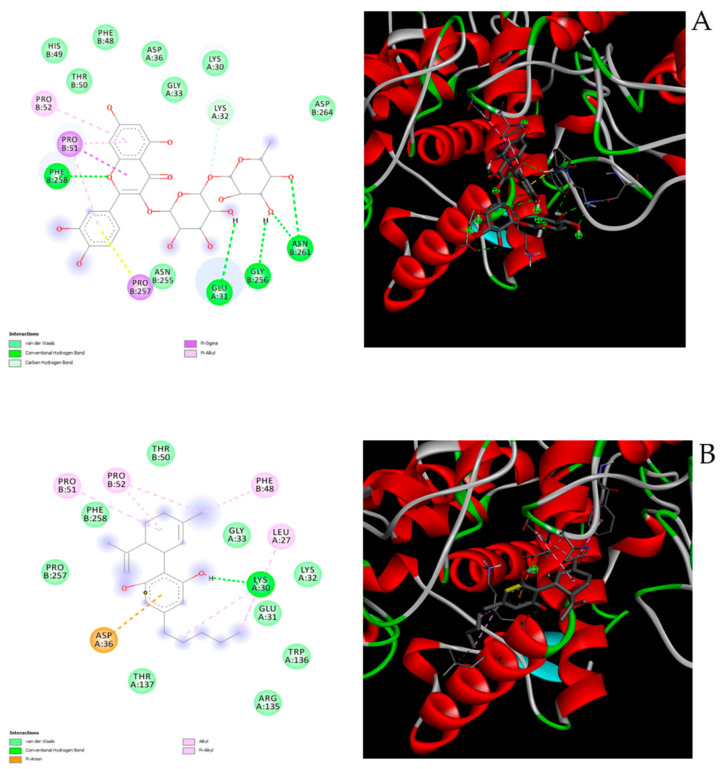

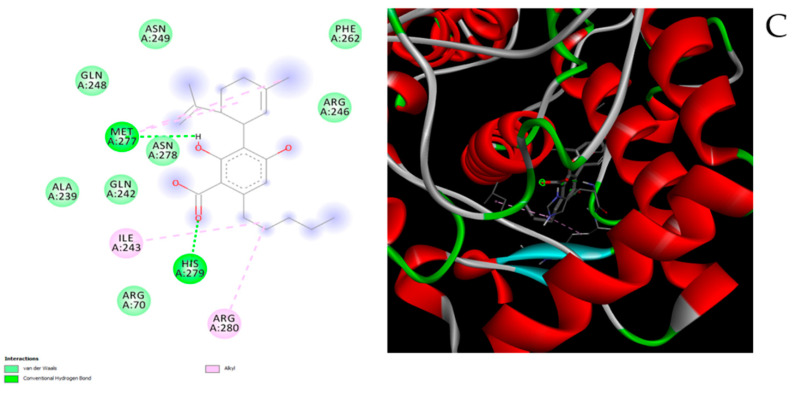
(**A**) Putative interactions between rutin and tyrosinase (PDB: 5I3B). Free energy of binding (ΔG) and affinity (Ki) are −8.3 kcal/mol and 0.8 µM, respectively; (**B**) putative interactions between cannabidiolic acid and tyrosinase (PDB: 5I3B). Free energy of binding (ΔG) and affinity (Ki) are −6.3 kcal/mol and 24.4 µM, respectively. (**C**) Putative interactions between cannabidiol and tyrosinase (PDB: 5I3B). Free energy of binding (ΔG) and affinity (Ki) are −6.2 kcal/mol and 28.9 µM, respectively.

On the other hand, the docking runs yielded a good affinity towards tyrosinase (0.8 µM) for the sole rutin (Figure 11A–C). On one side, this is consistent with the concentration-independent reduction in skin l-dopa turnover, following extract treatment. On the other side, the reduction in l-dopa depletion in skin specimens could also be related to intrinsic scavenging effects induced by the extract itself, as partially suggested by the measured antiperoxidase activity. Overall, our findings of antioxidant/anti-inflammatory effects induced by water hemp extract in in vitro and ex vivo models of skin injury support further studies aimed to validate the use of the present extract in dermatological formulations for counteracting skin inflammatory disorders, including hyperpigmentation.

## 4. Conclusions

In conclusion, the present findings highlight the potential of water extracts from industrial hemp inflorescences as skin protective agents. Additionally, the present study also yields a detailed description of the secondary metabolite composition of the hemp water extract that in addition to displaying appreciable amounts of phenolic compounds, is also relatively rich in cannabidiol and cannabidiolic acid. In this context, the present study supports an improvement of industrial hemp chain production, with particular regard to the byproducts constituted by female inflorescence, with promising applications in nutraceutical and cosmeceutical formulations.

## Figures and Tables

**Table 1:** Factors and levels selected for the design of experiment.

Independent Variables	Levels	
	−1	1
Time (min)	5	60
TEMPERATURE (°C)	25	80
UAE Power ^a^	1	5
Solid/liquid (mg/mL)	10	60

^a^ UAE power range automatically set by producer in 5 levels (20–120 W).

**Table 2:** Experimental design matrix with coded variables and experimental data for total polyphenols, total flavonoids and total tannins.

Variables				Experimental Results					
Time (min)	TEMP (°C)	Power	Solid/Liquid (mg/mL)	TPC	SD	TFC	SD	TTC	SD
60	80	3	35	0.280	0.010	0.158	0.01	0.328	0.025
5	80	3	35	0.248	0.022	0.128	0.009	0.304	0.036
60	52.5	3	60	0.363	0.020	0.191	0.007	0.366	0.024
5	52.5	3	60	0.390	0.038	0.187	0.007	0.448	0.015
60	52.5	3	10	0.390	0.010	0.070	0.008	0.141	0.010
32.5	80	3	10	0.129	0.013	0.074	0.004	0.180	0.033
32.5	80	5	35	0.283	0.002	0.157	0.006	0.307	0.015
32.5	25	5	35	0.280	0.010	0.121	0.002	0.242	0.007
5	52.5	5	35	0.258	0.012	0.115	0.004	0.277	0.027
32.5	52.5	1	60	0.371	0.024	0.213	0.004	0.412	0.012
32.5	52.5	1	10	0.123	0.007	0.052	0.004	0.147	0.017
32.5	25	1	35	0.288	0.045	0.139	0.009	0.289	0.018
5	52.5	1	35	0.272	0.025	0.131	0.003	0.255	0.020
32.5	25	3	10	0.110	0.006	0.044	0.002	0.105	0.009
32.5	52.5	3	35	0.285	0.012	0.118	0.004	0.316	0.033
5	52.5	3	10	0.108	0.002	0.059	0.004	0.145	0.011
60	52.5	5	35	0.284	0.005	0.129	0.006	0.280	0.008
32.5	25	3	60	0.427	0.046	0.222	0.001	0.400	0.035
60	52.5	1	35	0.347	0.013	0.146	0.002	0.287	0.015
32.5	80	3	60	0.441	0.024	0.231	0.002	0.463	0.014

Run sequence was conducted randomly. TPC: total polyphenols content expressed as GAE (mg/g); TFC: total flavonoids content expressed as rutin equivalents; TTC: total tannins content expressed as tannic acid equivalents.

**Table 3:** Gradient elution of HPLC-DAD.

Time (min.)	Flow (mL/min)	%A	%B
3	0.35	98	2
6		75	25
10		50	50
14		5	95
17		5	95
17.5		98	2
30		98	2

**Table 4:** Wavelengths of quantification and retention times related to the investigated phenolic compounds.

Standard		*m/z*	Wavelength (nm)	Retention Time (min)
1	Vanillic acid	167.1	222	17.4
2	Ferulic acid	193.1	222	18.2
3	Rutin	611.5	222	18.7
4	Naringenin	271.2	222	20.1

**Table 5:** Gradient elution of HPLC-UV-MS.

Time (min)	Flow (mL/min)	%A	%B
0	0.750	32.5	67.5
0.5	0.750	32.5	67.5
14	0.750	7	93
22	0.750	7	93
22.1	1.05	32.5	67.5
28	1.05	32.5	67.5
28.1	0.750	32.5	67.5
30	0.750	32.5	67.5

**Table 6:** Mass to charge ratios (*m/z*) and retention times related to the investigated terpenophenolic compounds.

Standard		*m/z*	Wavelength (nm)	Retention Time (min)
1	CBDA	357.3	230	9.9
2	CBGA	343.3–260.1	230	10.4
3	CBG	317.3–234.1–193.1	230	10.7
4	CBD	315.2	230	11.1
5	CBN	311.3–293.25	230	14.4
6	THC-d3	318.9; [315.2: extract pool of THC]	230	16.0
7	CBC	315.3–259.13–193.13	230	17.5
8	THCA	341.3	230	18.1

**Table 7:** Total phenols, flavonoids, tannins content and antiperoxidase activity of water hemp extract.

Total Phenols (mg GA/g Extract)	Total Flavonoids (mg RU/g Extract)	Total Tannins (mg TA/g Extract)	Antiperoxidase Activity (EC_50_: mg/mL)
7.12 ± 0.35	3.92 ± 0.08	5.74 ± 0.42	4.12 ± 0.37

GA: Gallic acid; RU: Rutin; TA: Tannic acid.

## Data Availability

The data presented in this study are available on request from the corresponding author.

## Supplementary Materials

The following are available online https://www.mdpi.com/2076-3921/10/1/44/s1.

## Sample Availability

Samples of the compounds are available from the authors.
