# Supplementary material for: Metabolomic Profile and Antioxidant/Anti-Inflammatory Effects of Industrial Hemp Water Extract in Fibroblasts, Keratinocytes and Isolated Mouse Skin Specimens
Source: Antioxidants (Basel). 2021 Jan 1;10(1):44. doi: 10.3390/antiox10010044 (PMC7823476; doi:10.3390/antiox10010044)
Supplement: Supplementary file 1 [file antioxidants-10-00044-s001.pdf]

S1. Estimated regression coefficient of the second order polynomial equations for RSM analysis of total phenols. total flavonoids and total tannins extraction (uncoded) from hemp inflorescences

Terms	Regression coefficient	Probability	Regression *p*-value	R^2
POLYPHENOLS				
Constant	0.158204	0.011	0.178	86.75%
Time (min)	0.00667764	0.144		
TEMP (°C)	0.00157144	0.811		
Power	0.0252778	1.000		
Solid/liquid (mg/mL)	0.00902893	0.008		
Time (min)*Time (min)	2.38261E-05	0.714		
TEMP (°C)*TEMP (°C)	1.10125E-05	0.866		
Power*Power	-0.00333582	0.788		
Solid/liquid (mg/mL)*Solid/liquid (mg/mL)	-8.60832E-07	0.991		
Time (min)*TEMP (°C)	-3.81034E-05	0.637		
Time (min)*Power	-2.22128E-04	0.750		
Time (min)*Solid/liquid (mg/mL)	-1.12649E-04	0.086		
TEMP (°C)*Power	0.000212877	0.810		
TEMP (°C)*Solid/liquid (mg/mL)	-1.76491E-06	0.975		
Power*Solid/liquid (mg/mL)	-2.63414E-04	0.828		
FLAVONOIDS				
Constant	0.0968723	0.001	0.002	98.08%
Time (min)	-5.89835E-04	0.352		
TEMP (°C)	-0.00241774	0.140		
Power	-0.0143970	0.454		
Solid/liquid (mg/mL)	0.00315363	0.000		
Time (min)*Time (min)	3.83401E-06	0.777		
TEMP (°C)*TEMP (°C)	2.17840E-05	0.154		
Power*Power	0.00194474	0.463		
Solid/liquid (mg/mL)*Solid/liquid (mg/mL)	1.04198E-05	0.531		
Time (min)*TEMP (°C)	1.22803E-05	0.473		
Time (min)*Power	-6.70833E-06	0.963		
Time (min)*Solid/liquid (mg/mL)	-2.40177E-06	0.836		
TEMP (°C)*Power	0.000120494	0.522		
TEMP (°C)*Solid/liquid (mg/mL)	-7.81568E-06	0.510		
Power*Solid/liquid (mg/mL)	-1.61419E-04	0.530		
TANNINS				
Constant	-0.0277443	0.000	0.001	98.50%
Time (min)	0.00159710	0.478		
TEMP (°C)	0.000904833	0.028		
Power	0.0180290	0.662		
Solid/liquid (mg/mL)	0.00861505	0.000		
Time (min)*Time (min)	-2.67590E-05	0.256		
TEMP (°C)*TEMP (°C)	-1.13031E-05	0.615		
Power*Power	-0.00528716	0.242		
Solid/liquid (mg/mL)*Solid/liquid (mg/mL)	-3.28284E-05	0.250		
Time (min)*TEMP (°C)	2.45179E-05	0.385		
Time (min)*Power	-1.30303E-04	0.586		
Time (min)*Solid/liquid (mg/mL)	-2.84848E-05	0.173		
TEMP (°C)*Power	0.000219153	0.477		
TEMP (°C)*Solid/liquid (mg/mL)	-4.42424E-06	0.815		
Power*Solid/liquid (mg/mL)	0.000123194	0.765		

S2. Optimized operative conditions and predictive effects on extraction of TPC, TFC and TTC

Response analyzed	Time (min)	TEMP (°C)	Power	Solid/liquid (mg/mL)	Predicted yield
TPC	60	25	1	60	0.4379
TFC	60	80	1	60	0.2479
TTC	25.5	80	3.7	60	0.4538
TPC*TFC*TTC	36.11	80	2.57	60	0.4019 (TPC)
					0.2211 (TFC)
					0.4451 (TTC)
TFC*TTC	25	80	5	60	0.3846 (TPC)
					0. 2163 (TFC)
					0.4456 (TTC)

S2. The response optimizer function was applied to single response (TPC, TFC or TTC) and to their compination. Finally, a combination of the significant TFC and TTC was tested and selected to define the operative conditions applied to extract preparation for experimental phytochemical and pharmacological investigations.

S3. A contour plot representation of interaction of critical terms, namely solid/liquid ratio, extraction time and temperature, on total polyphenols (A), flavonoids (B) and tannins (C) extraction.

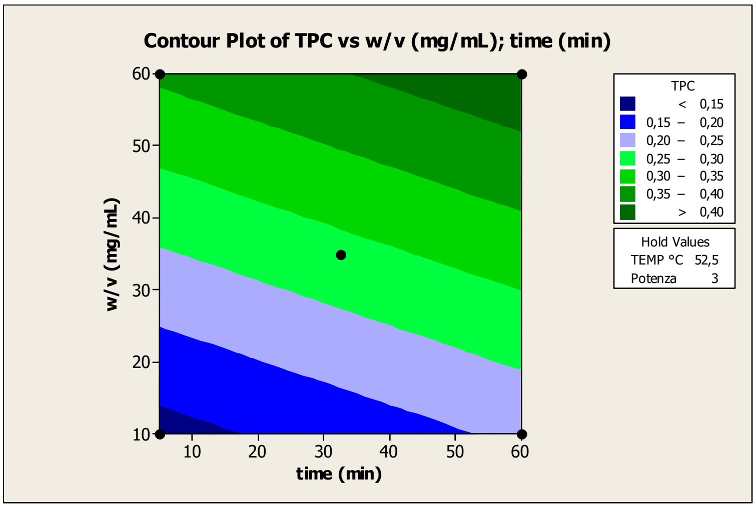

A

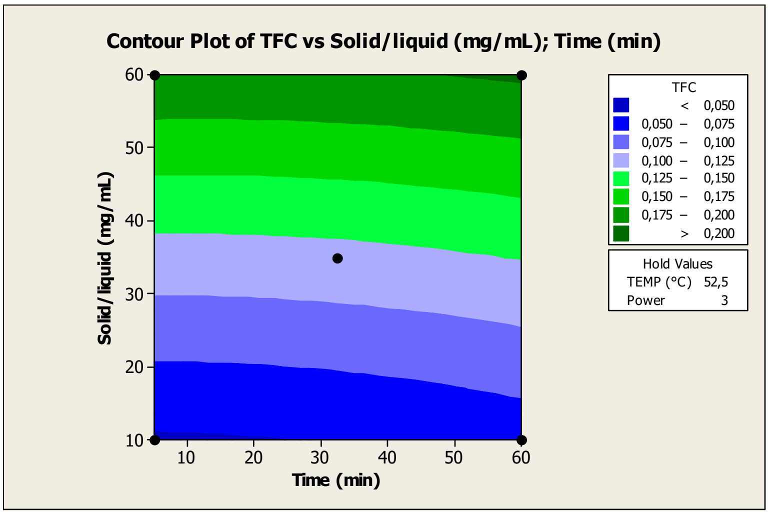

B

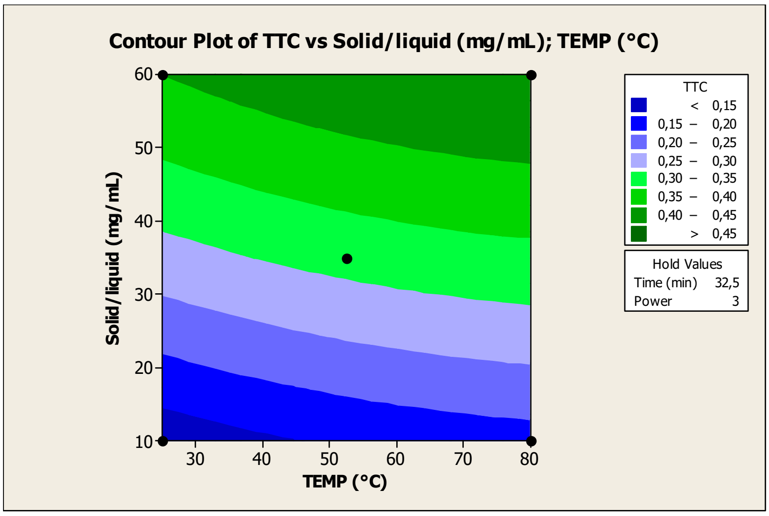

C
